# Comparison of Standard of Care with or Without a PD-1/PD-L-1 Inhibitor for the Treatment of Multiple Myeloma: A Systematic Review and Meta-Analysis of Phase II and III Randomized Controlled Trials

**DOI:** 10.3390/cancers17233730

**Published:** 2025-11-21

**Authors:** Zain Alsaddi, Osama Younis, Ghena Khasawneh, Yara Shatnawi, Nausheen Ahmed, Zahra Mahmoud Jafari, Muhammad Umair Mushtaq, Al-Ola Abdallah, Shebli Atrash, Barry Paul

**Affiliations:** 1 Department of Internal Medicine, King Hussein Military Hospital, Jordanian Royal Medical Services, Amman 11855, Jordan; zain.salsaddi@gmail.com; 2 School of Medicine, University of Jordan, Amman 11942, Jordan; 3 Taussig Cancer Center, Cleveland Clinic, Cleveland, OH 44106, USA; ghena.khasawneh@yahoo.com; 4 Department of Gastroenterology and Hepatology, Baylor College of Medicine, Houston, TX 77030, USA; 5 Division of Hematologic Malignancies & Cellular Therapeutics, University of Kansas Medical Center, Westwood, KS 66205, USAzmahmoudjafari@kumc.edu (Z.M.J.);; 6 Department of Hematologic Oncology & Blood Disorders, Levine Cancer Institute, Atrium Health Wake Forest University School of Medicine, Charlotte, NC 27103, USA

**Keywords:** immune checkpoint inhibition, multiple myeloma, PD-1, immunotherapy

## Abstract

Background: Despite recent advances in the treatment of multiple myeloma (MM), the disease remains incurable. Agents targeting the programmed death-1 (PD-1) axis have become key treatments for many cancers; however, monotherapies with PD-1/PD-L-1 inhibitors (PD-1i) have shown a limited efficacy in MM patients. We conducted a meta-analysis to evaluate the safety and efficacy of PD-1i in combination with standard of care (SOC) therapies for MM. Methods: A systematic search identified phase II and III randomized clinical trials involving MM patients treated with PD-1i, using terms such as “Relapsed Multiple Myeloma” and “PD-1 inhibitors.” The search included trials published in English after 2016 using the PubMed, CINAHL, and Cochrane databases. Data were analyzed using RStudio v4.4.2, with survival data assessed using Kaplan–Meier methods and Cox proportional hazards analysis. Results: Our search yielded 19 total trials, of which 5 (representing 889 unique patients) met the criteria for inclusion. A total of 488 patients received PD-1i + SOC, and 401 received SOC alone. The median progression-free survival (PFS) was 6.26 months for the PD-1i + SOC group and 7.34 months for the SOC alone group (HR, 1.2; 95% CI: 0.95–1.41; *p* = 0.14). The median overall survival (OS) was 22.49 months for PD-1i + SOC compared to 24.38 months for SOC alone (HR, 1.11; 95% CI: 0.87–1.42, *p* = 0.36). The PD-1i group showed a higher incidence of all-grade pneumonia (RR = 1.44, 95% CI: 1.03–2.02, *p* = 0.03), grade ≥ 3 neutropenia (RR = 1.36, 95% CI: 1.00–1.85, *p* = 0.05), immune-mediated adverse events (RR = 10.1, 95% CI: 0.38–271.1, *p* = 0.17), and grade ≥ 3 irAEs (RR = 9.59, 95% CI: 0.62–148.8, *p* = 0.11). Conclusion: This analysis highlights the limited efficacy and increased toxicity of adding PD-1i to SOC in MM patients. Further research is needed to explore whether targeting the PD-1/PD-L1 axis in combination with novel therapies can safely enhance the therapeutic efficacy in MM patients.

## Simple Summary

This study explores the role of immunotherapy, a form of drug treatment that utilizes one’s own immune system to fight tumor cells, in multiple myeloma. We investigated all the clinical trials published on this topic and identified that the use of immunotherapy in multiple myeloma is not associated with an increased survival, while resulting in a higher burden of adverse events such as pneumonia (lung infections) and skin rash. Thus, we conclude that immunotherapy requires a more rigorous patient selection in order to find a subset with a response that outweighs the burden of adverse events.

## 1. Introduction

Multiple myeloma (MM) is a common hematological cancer, accounting for approximately 20% of hematologic malignancies, with an incidence of approximately 4 cases per 100,000 individuals in the U.S. [1]. Recent advancements in treatments such as immunomodulatory drugs, monoclonal anti-CD38 antibodies, and CAR-T cells have led to significant improvements in survival; however, MM remains incurable, and patients inevitably experience relapses [2,3].

Immune checkpoint blockade (ICB) has become integral to the treatment landscape of solid tumors and some hematological cancers such as Hodgkin lymphoma. Immune checkpoints inhibit the cytotoxic capacity of CD8+ T cells, decrease T cell proliferation, and inhibit TH1 cytokine secretion. The most prominent checkpoint axis is the PD-1/PD-L1 axis. PD-1 is a molecule characteristically expressed by monocytes, T cells, NK cells, and activated B cells. Its ligand, PD-L1, is expressed on antigen-presenting cells such as macrophages and dendritic cells. This axis is integral to controlling the immune system and ensuring an appropriate immune response by decreasing the number of autoreactive T cells. However, this axis can also be utilized by cancer cells that up-regulate PD-L1, limiting the extent of immune-mediated tumor killing. The inhibition of this axis results in an increased immune infiltration, reactivation of exhausted CD8+ T cells, and increased anti-tumor immunity [4].

Immune dysregulation is crucial for MM development. The immune microenvironment of MM is notoriously immunosuppressive, with a quantitative decrease in myeloma-specific lymphocytes, such as NKT cells and CD8+ T cells, coupled with an increased infiltration of T-regulatory cells (Tregs) and myeloid-derived suppressor cells (MDSC), associated with an increase in PD-1/PD-L1 expression in MM cells and multiple immune cell types [5]. Yousef et al. showed that malignant plasma cells have a higher expression of PD-L1 when compared to other pre-malignant plasma cell dyscrasias or normal plasma cells [6]. Moreover, studies have shown that PD-L1 is overexpressed in MDSCs and plasmacytoid dendritic cells (pDCs), and the subsequent blockade of the PD-1/PD-L1 axis leads to a reversal of the immunosuppressive effect of MDSCs and increased generation of cytotoxic T cells by pDCs, which ultimately leads to an increased anti-tumor immunity [7,8].

However, despite sufficient biological and pre-clinical evidence supporting the notion of PD-1/PD-L1 blockade, the efficacy of such treatment modalities in MM remains questionable [9].

In this study, we performed the first meta-analysis to evaluate both the efficacy and safety of adding PD-1/PD-L1 inhibitors to the standard treatment backbones used in the treatment of MM. We assessed multiple outcomes including survival, patient response, and adverse events.

## 2. Methodology

### 2.1. Search Criteria

We designed this study to assess the toxicity and efficacy of PD-1/PDL-1 inhibitors in patients with newly diagnosed or relapsed MM. We searched three online databases, PUBMED, CINAHL, and Cochrane Library, for related publications using the following search terms: “Relapsed Multiple Myeloma” with any of the terms “PDL-1 inhibitors” OR “PDL-1” OR “programmed death ligand 1 inhibitors”. Restriction to the English language was applied. This study was not registered in PROSPERO.

### 2.2. Inclusion and Exclusion Criteria

The eligibility criteria for inclusion in our meta-analysis required adherence to specific methodological standards. Trials had to meet the following requirements: (1) published after 2016; (2) phase II and III randomized trials; (3) study populations comprising patients with newly diagnosed or relapsed MM; (4) participants aged 18 years or older; (5) published in English; (6) provided adequate data on key survival outcomes, including progression-free survival (PFS) and overall survival (OS), with a minimum of 6 months follow-up; and (7) provided adequate incidence of adverse events among patient populations. Abstracts, editorials, case reports, case series, review articles, case–control studies, and retrospective cohort studies were excluded.

### 2.3. Data Extraction and Quality Assessment

Three investigators screened the titles and abstracts of potentially relevant studies using the spreadsheet software “Microsoft Excel”. The full texts of the relevant studies were retrieved and further reviewed by three investigators. The extracted data were tabulated using Microsoft Excel and reviewed by a third investigator. We extracted the following data: author, study population, median lines of treatment, number of patients, median age, study period, treatment regimens (arms), incidence of immune-related adverse events, neutropenia, URTIs, and pneumonia.

Individual patient survival data (IPD) were extracted from Kaplan–Meier plots using the “DigitizeIt v2.6” software. Subsequently, the IPD were pre-processed and analyzed using the modified iKM algorithm, which applies multiple quality control steps, such as outlier detection and protocols, to prevent underestimation. We utilized the IPD from the KM method developed by Liu et al. [10].

We defined the outcomes of interest as objective response rate (ORR), complete response rate (CR), very good partial response rate (VGPR), partial response rate (PR), stable disease rate (SD), progression-free survival (PFS), overall survival (OS), and incidences of anemia, neutropenia, thrombocytopenia, pneumonia, diarrhea, and rash.

Publication bias was assessed using funnel plots with the zones of statistical significance highlighted. Publication bias funnel plots can be seen in Figures S4–S6.

Finally, quality assessment was conducted using Cochrane’s Risk of Bias Tool 2 (ROB2), which was created to assess randomized controlled trials in the following domains: randomization process, deviations from intended outcomes, missing outcome data, measurement of outcomes, and selective reporting of data. The quality assessment outcomes are summarized in Figure S1.

### 2.4. Statistical Analysis

Meta-analysis was conducted using the “Meta” package on R version 4.4.2. Heterogeneity was considered significant if I^2^ was ≥50% or the *p*-value was <0.10. A fixed-effects model using the Mantel–Haenszel method was adopted for outcomes with no heterogeneity. Conversely, a random-effects model using the DerSimonian and Laird method was used for heterogeneous outcomes. The hazards ratio (HR) was used for survival outcomes, while relative risk (RR) was used for response and incidence of adverse events. The 95% confidence intervals (CI) were reported for both effect measures. We also conducted a sensitivity analysis using leave-one-out plots with the same stipulations mentioned above, which can be found in Figures S3 and S4. Publication bias was assessed using statistically bounded funnel plots, which are found in Figures S5 and S6.

## 3. Results

### 3.1. Search Results

**Figure 1:**
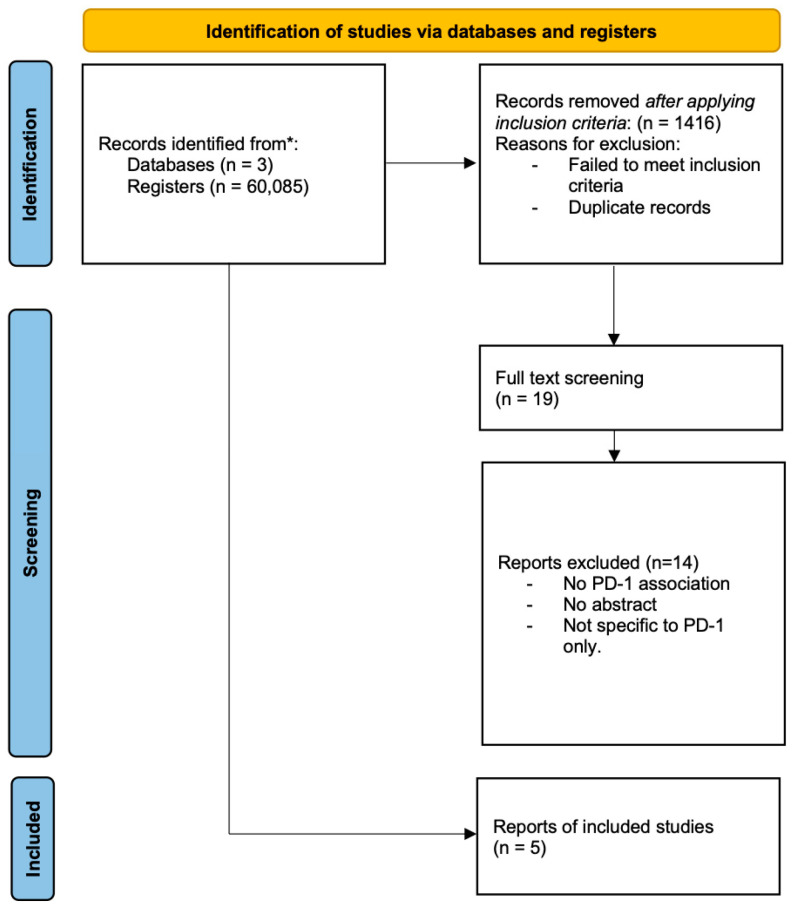
PRISMA workflow for article selection.

We identified a total of 60,088 records from the database search. The initial de-duplication and adherence to inclusion criteria resulted in 1416 records for title and abstract screening. After title and abstract screening, a total of 19 articles were left for full-text screening. Full-text screening identified 5 papers that were eligible for analysis, while the other 14 were excluded for the reasons mentioned in the Section 2. Figure 1 and Table 1 show the PRISMA workflow for screening and the characteristics of the five included articles, respectively.

### 3.2. Efficacy

A total of 889 patients were included in this meta-analysis. According to the inclusion criteria of each trial, patients were required to have sufficient hepatic, renal, and hematologic functions. A total of 488 patients were treated with PD-1i + SOC, and 401 patients were treated with SOC alone (Table 2).

#### 3.2.1. Survival Outcomes

**Figure 2:**
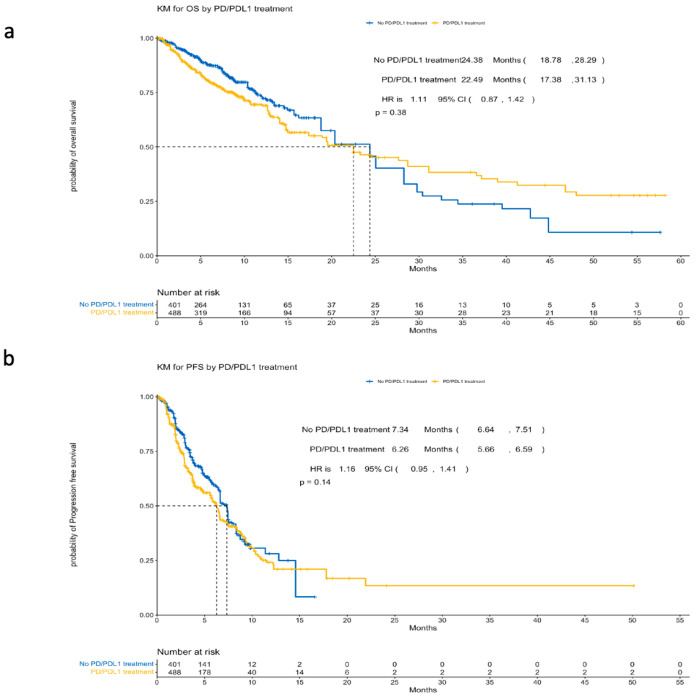
Kaplan–Meier plots of reconstructed individual patient data. (**a**) Overall survival; (**b**) progression-free survival.

There was no clinical difference in the median overall survival (OS) between PD-1i + SOC (22.49 months) and SOC (24.38 months) (Table 2). The findings indicated that the addition of PD-1i to SOC resulted in minimal changes in the overall survival (HR = 1.11, 95% CI: 0.87–1.42, *p* = 0.36; Figure 2a). A detailed report on the overall survival statistics based on the study cohorts is shown in Table 3.

Progression-free survival (PFS) data were obtained from four trials. Using Kaplan–Meier survival analysis, the median PFS for the PD-1i plus SOC group was 6.26 months, compared to 7.34 months for SOC alone (HR = 1.16, 95% CI: 0.95–1.41, *p* = 0.14; Figure 2b).

#### 3.2.2. Response Rate Outcomes

**Figure 3:**
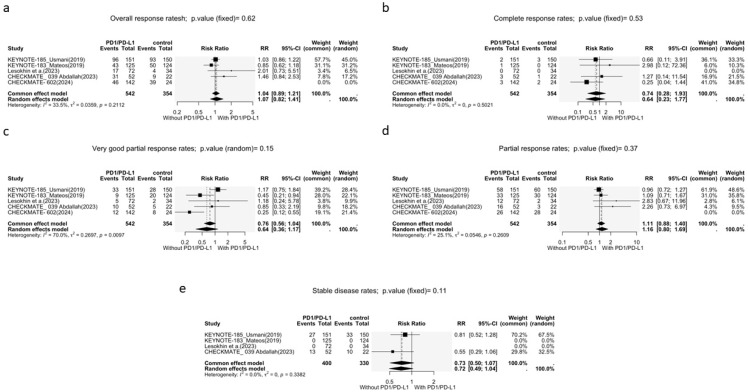
Forest plots of MM response rates. (**a**) Overall response rate; (**b**) complete response rates; (**c**) very good partial response rates; (**d**) partial response rates; (**e**) stable disease rates.

All five trials provided data on the overall response rate (ORR). The analysis revealed no significant difference between the two groups, with a relative risk (RR) of 1.04 (95% CI: 0.81–1.26, *p* = 0.21; Figure 3a) based on a common-effects model. The heterogeneity was not significant (I^2^ = 33.5%, *p* = 0.21).

All five trials also reported data on complete response rates (CR), very good partial response rates (VGPR), and partial response rates (PR); all but CheckMate-602 reported stable disease rates (SD); CR showed no significant difference between PD-1i’s + SOC versus SOC alone (RR = 0.74, 95% CI: 0.28–1.93; Figure 3b) based on a common-effects model (I^2^ = 0%, *p* = 0.5021). VGPR showed similar results, with an RR of 0.76 (95% CI: 0.56–1.04, *p* = 0.15; Figure 3c). The heterogeneity was significant for VGPR; thus, a random-effects model was used (I^2^ = 70%, *p* < 0.01). No differences were observed between the two treatments in terms of PR (RR = 1.11, 95% CI: 0.88–1.40, *p* = 0.37; Figure 3d) and SD (RR = 0.73, 95% CI: 0.50–1.07, *p* = 0.11; Figure 3e). Neither PR (I^2^ = 25.1%, *p* = 0.26) nor SD (I^2^ = 0%, *p* = 0.34) showed significant heterogeneity; thus, a fixed-effects model was used.

### 3.3. Safety

This meta-analysis documented a range of treatment-related adverse events (TRAEs) associated with PD-1i.

#### 3.3.1. Immune-Related Adverse Events

**Figure 4:**
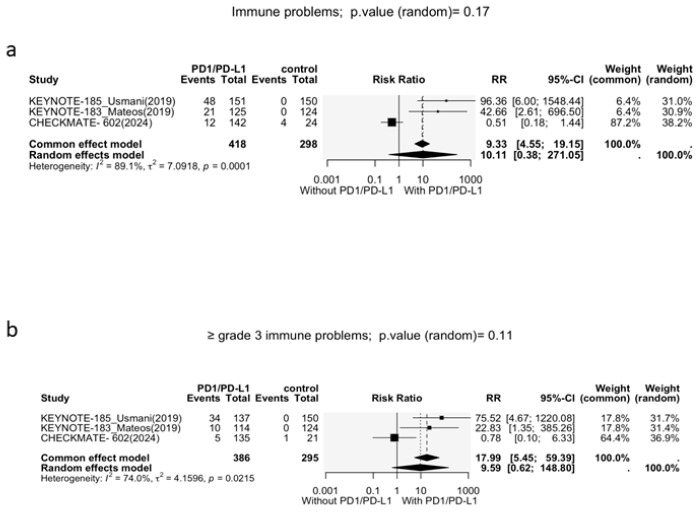
Forest plots of immune-related adverse events. (**a**) All-grade immune-related adverse events; (**b**) grade 3–4 immune adverse events.

Three studies reported cumulative immune-related adverse events (irAEs). A statistically insignificant trend of increased irAE risk was observed in patients receiving PD-1i + SOC (RR = 10.1, 95% CI: 0.38–271.1, *p* = 0.17; Figure 4a). A large heterogeneity was observed between the studies (I^2^ = 89.1%, *p* < 0.01); thus, a random-effects model was employed.

This trend persisted when assessing adverse events of grade 3 or higher. PD-1i + SOC showed a statistically non-significant increased risk of irAEs compared to SOC alone (RR = 9.59, 95% CI: 0.62–148.8, *p* = 0.11; Figure 4b). The heterogeneity was significant (I^2^ = 74%, *p* < 0.01), necessitating a random-effects model.

It is important to note that statistical non-significance was mainly driven by CheckMate-602. The removal of CheckMate-602 yielded a significant increase in the risk of all grades (RR = 69.50, 95% CI: 9.72–496.85, *p* < 0.001; Figure S2a), and grade 3/4 immune-related adverse events (RR = 41.91, 95% CI: 5.77–304.33, *p* < 0.001; Figure S3a). The heterogeneity also dropped to 0% after the removal of CheckMate-602.

#### 3.3.2. Other Adverse Events

We also assessed the risk of infections and blood, gastrointestinal, and skin adverse events in patients taking PD-1i + SOC versus SOC alone.

##### Neutropenia

**Figure 5:**
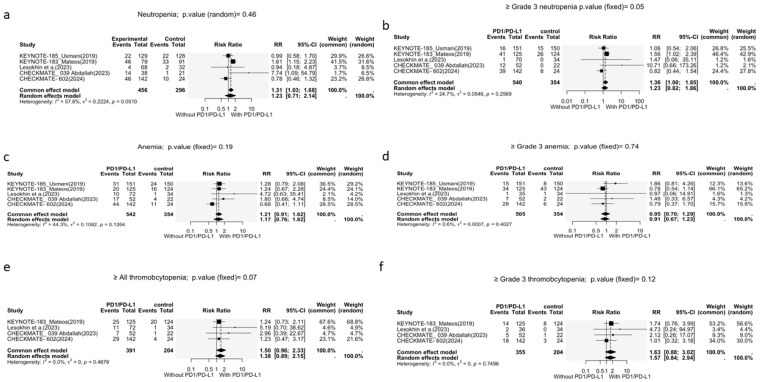
Forest plots of hematological adverse events. (**a**) All-grade neutropenia; (**b**) grade 3–4 neutropenia; (**c**) all-grade anemia; (**d**) grade 3–4 anemia; (**e**) all-grade thrombocytopenia; (**f**) grade 3–4 thrombocytopenia.

Our analysis showed no significant association between PD-1i and the incidence of all-grade neutropenia (RR = 1.23, 95% CI: 0.71–2.14, *p* = 0.46; Figure 5a). A random-effects model was used because of the significant heterogeneity (I^2^ = 57.6%, *p* = 0.051).

However, the addition of PD-1i to SOC increased the risk of grade 3 or higher neutropenia (RR = 1.36, 95% CI: 1.00–1.85, *p* = 0.05; Figure 5b). A fixed-effects model was used because of the non-significant heterogeneity (I^2^ = 24.7%, *p* = 0.26).

##### Anemia

All five studies reported anemia as an adverse event. No significant difference between PD-1i + SOC and SOC alone was observed (RR = 1.21, 95% CI: 0.91–1.62, *p* = 0.19; Figure 5c).

The same results were observed when comparing the risk of grade 3 anemia (RR = 0.95, 95% CI: 0.70–1.29, *p* = 0.74; Figure 5d). The heterogeneity was not significant in either analysis (I^2^ = 44.3%, *p* = 0.13; or I^2^ = 0.6%, *p* = 0.40).

The removal of CheckMate-602 showed an increased risk of all-grade anemia (RR = 1.43, 95% CI: 1.01–2.02, *p* = 0.043; Figure S2c), while simultaneously nullifying all previous heterogeneities. No statistically significant difference was observed in grade 3/4 anemia.

##### Thrombocytopenia

Four studies reported the incidence of thrombocytopenia. Our analysis showed no significant difference in the incidence of all grades of thrombocytopenia between the treatment arms (RR = 1.50, 95% CI: 0.96–2.33, *p* = 0.07; Figure 5e). The same trend was observed for grade 3 and 4 thrombocytopenia (RR = 1.63, 95% CI: 0.88–3.02, *p* = 0.12; Figure 5f). A fixed-effects model was used for both outcomes, as neither showed a significant heterogeneity (I^2^ = 0.0%, *p* = 0.49) (I^2^ = 0.0%, *p* = 0.75, respectively).

##### Pneumonia

**Figure 6:**
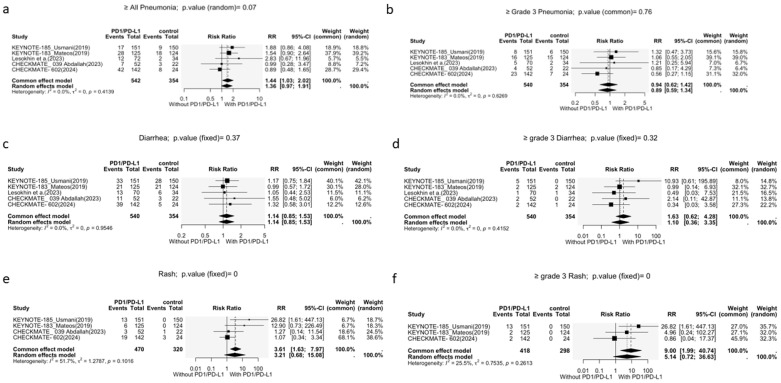
Forest plots of organ-specific adverse events. (**a**) All-grade pneumonia; (**b**) grade 3–4 pneumonia; (**c**) all-grade diarrhea; (**d**) grade 3–4 diarrhea; (**e**) all-grade rash; (**f**) grade 3–4 rash.

Pneumonia was reported as an adverse event in all studies. A significant association between the addition of PD-1i and the risk of all-grade pneumonia was observed (RR = 1.44, 95% CI: 1.03–2.02, *p* = 0.03; Figure 6a). However, the addition of PD-1i did not increase the risk of grade 3/4 pneumonia (RR = 0.94, 95% CI: 0.62–1.42, *p* = 0.76; Figure 6b). No heterogeneity was present for both outcomes.

##### Diarrhea

Diarrhea was reported in all five studies. No significant association was found between the incidence of all-grade diarrhea and the addition of PD-1i to SOC (RR = 1.14, 95% CI: 0.85–1.53, *p* = 0.37; Figure 6c). A fixed-effects model was used because of the absence of heterogeneity (I^2^ = 0.0%, *p* = 0.95).

Furthermore, a stratified analysis of grade 3 and 4 diarrhea also showed no significant association with PD-1i (RR = 1.63, 95% CI: 0.62–4.28, *p* = 0.32) using a fixed-effects model (I^2^ = 0.0%, *p* = 0.42).

##### Rash

A total of four articles reported the incidence of all-grade rash, while three reported the incidence of grade 3/4 rash. The addition of PD-1i to SOC did not significantly increase the risk of all-grade rash (RR = 3.21, 95% CI: 0.68–15.08, *p* = 0.14; Figure 6e); however, a significant increase in risk was observed for grade 3/4 rash (RR = 9.00, 95% CI: 1.99–40.74, *p* < 0.01; Figure 6f). The heterogeneity was significant for all-grade rash (I^2^ = 51.7%, *p* = 0.10) but insignificant for grade 3/4 rash (I^2^ = 25.5%, *p* = 0.26).

## 4. Discussion

To improve clinical decision-making in MM, this meta-analysis evaluated the efficacy and safety of PD-1i when integrated into SOC regimens for MM, including adult patients (aged 19 years and older) with newly diagnosed or relapsed multiple myeloma who received PD-1i combined with standard-of-care regimens. Although checkpoint inhibitors have achieved success in solid and blood tumors, such as NSCLC and Hodgkin lymphoma, their efficacy in MM has not yet been confirmed [4].

A total of 900 patients across five RCTs were included; the meta-analysis failed to identify a significant difference in the overall response rate (RR = 1.01; 95% CI: 0.81–1.26), with no pertinent signals when looking at the complete response, partial response, or stable disease rate. Furthermore, no significant difference in the overall survival (HR = 1.11; 95% CI: 0.87–1.42; *p* = 0.38) was observed between the PD-1i + SOC and SOC groups. The findings remained consistent across all studies, regardless of RRMM or newly diagnosed MM population.

Minor improvements in PFS were observed in smaller or early-stage studies; however, these effects were absent in larger randomized trials. For example, pembrolizumab failed to show OS or PFS benefits in KEYNOTE-183 and KEYNOTE-185 trials, resulting in the premature discontinuation of the trials due to safety concerns. Similarly, CHECKMATE-602 showed a lower overall RR in the nivolumab-containing arms (Nivo-Pd: 48%, NE-Pd: 42%) compared to SOC (Pd: 55%) [15]. Furthermore, our IPD analysis included 889 out of the 900 patients and did not show significant results for both PFS and OS.

In early-phase research, for instance, a study by Lesokhin et al. reported promising results with checkpoint blockades, particularly in combinations involving cemiplimab and isatuximab. Nonetheless, these findings are preliminary validations through larger controlled settings. Variabilities in the study design and patient characteristics complicate interpretation and limit external validity [12].

PD-1/PD-L1 blockade was associated with a higher overall burden of treatment-related toxicities: irAEs showed a large numerical difference between treatment and control arms when pooling all studies, and became statistically significant once CHECKMATE-602 was excluded. Specific organ system events also trended upward, including all-grade pneumonia (significantly higher), grade ≥ 3 neutropenia (borderline significant), all-grade thrombocytopenia and anemia (both non-significant overall, but all-grade anemia became significant after CHECKMATE-602 removal), and grade ≥ 3 rash (significantly higher); in contrast, diarrhea rates were unaffected. These additive toxicities translated into higher treatment discontinuation rates and reinforced concerns about the tolerability of checkpoint inhibitors in MM patients, as highlighted in KEYNOTE-185 and CHECKMATE-039. This is most likely due to the over-activation of effector T cells against normal host tissues that normally express PD-L1 to induce immunological tolerance. This function is utilized by various tissues in the body, such as the heart, pancreas, placenta, vascular endothelium, liver, lung, and skin [16]. This is not limited to MM, as the use of PD-1i’s in the treatment of non-small-cell lung cancer shows a similar safety profile with an increased risk of pneumonitis/pneumonia, skin rash, and endocrine side effects [17].

The heterogeneity introduced by CHECKMATE-602 is an area of interest. We believe that the early halting of all in-class trials is the most likely culprit of such divergence. Early signals of increased death in the PD-1 arms of Keynote-183 and -185 led to the temporary halting of all PD-1/MM trials. Subsequent amendments to protocols were approved by the FDA, and the resumption of trials was met with an increased vigilance to adverse events. This increased observation most likely led to the earlier administration of corticosteroids, earlier treatment stoppages, and resulted in the overall decrease in the difference in outcomes between the Nivo-Pd and Pd arms in the CHECKMATE-602 trial.

CD4+ and CD8+ T cells are the dominant host effector cells against myeloma, as shown by [18,19] via in vivo testing in murine models. This is further reinforced by the outstanding success of CAR-T cells in treating MM. Thus, the absence of significant clinical benefits with the use of PD-1is, which are poised to unlock the full anti-tumor potential of host T cells, is interesting. The most likely explanation for this phenomenon is the severely immunosuppressive microenvironment within the bone marrow, and specifically within the malignant stroma of MM. In particular, the extra-cellular matrix (ECM) of MM has been shown to up-regulate multiple adherence molecules such as VLA-4 and Integrin B7. This facilitates the binding to fibronectin and the subsequent activation of p27 and nuclear factor κB, leading to a phenomenon called cell adhesion-mediated drug resistance (CAM-DR) to conventional chemotherapy [20,21]. CAM-DR reversal via targeting VLA-4 has been proven feasible in pre-clinical studies and might be useful in overcoming ECM-based features of MM treatment resistance [22]. Furthermore, studies have shown that MM tends to have a correlation with CD4+ CD25+ T cells (Treg), which are known to suppress CD8+ T cells by outcompeting them for stimulatory IL-2 signals [23]. An increased Treg frequency has been shown to result in a poorer PFS in patients with newly diagnosed MM, especially when considering their frequency in relation to the abundance of effector CD4+ T cells. Treg frequency is also positively correlated with PD-1 expression status in CD8+ and CD4+ effector T cells in the BM, with the same subset of CD4+ PD-1+ T cells co-expressing LAG-3/CTLA-4, indicating a more immune-suppressed microenvironment, with the up-regulation of multiple different immune checkpoints within MM patients that have PD-1 expressing effector T cells [24]. Other key players in the immunosuppressive microenvironment of MM are plasmacytoid dendritic cells (pDCs). Normal dendritic cells are antigen-presenting cells that activate and initiate effector T cell proliferation and differentiation; however, studies have shown that pDCs found in the bone marrow of MM patients lack the antigen-presenting capacity of normal BM dendritic cells. In addition to that, MM cells co-cultured with pDCs showed an increased proliferative capacity, with pDCs in MM patients being more abundant in the bone marrow (the main site of MM proliferation) when compared to peripheral blood [25]. A study by Ray et al. also showed that an inhibition of the PD1-PD-L1 axis did not affect the ability of pDCs to induce MM proliferation and survival; however, they did report that PD-L1 inhibition resulted in pDC-MM cell interactions that up-regulate PDL1 expression in both cell types and generate MM-specific cytotoxic T cells capable of eliminating previously drug-resistant MM cells [8]. This pro-proliferative sign is not specific to pDCs, as mesenchymal stem cells (MSCs) are another cell type that support multiple myeloma proliferation directly via the suppression of T cells through the PD-1 axis, and others such as the DKK1+ MM cells and Il-6-producing undifferentiated MSCs [26,27,28].

Recent studies have also shown that immune checkpoint blockades (specifically PD-1) might be better situated in scenarios where the patients are receiving T cell-based therapies such as CAR-T or Bispecific antibodies. A recent study by Verkleij et al. and colleagues showed that the ex vivo killing capacity of Talquetamab or Teclistimab was decreased in patients whose BM contains PD-1+ CD4+ T cells or CTLA4+ T cells [29]. These results are further corroborated by Mishra et al. and Dhodapkar, who discovered that a similar trend was observed in BCMA-targeting CAR-t cells. MM patients who relapsed post-CAR T had significant subsets of PD1+ T cells, with some expressing other co-inhibitory molecules such as TIGIT, KLGR1, and BAFF [30,31]. This compliments what is previously discussed in the literature and shown in our results. PD-1 inhibition with SOC alone is insufficient; instead, it needs rigorous patient selection criteria where its role in decreasing resistance to cellular therapies might be of clinical relevance.

This study has several limitations. First, the results were heavily influenced by two large trials, KEYNOTE-183 and -185, which comprised over 60% of the sample. Second, only five RCTs were included, and the statistical power was limited. Third, the follow-up durations were insufficient for evaluating long-term survival outcomes and late-onset adverse events. Furthermore, the small number of studies did not allow us to create patient subgroups that would be meaningful; however, this does not limit the speculation of possible treatment subgroups by referencing pre-clinical and non-randomized trials for future trials. In addition, language restrictions on English publications reduced the total number of accessible studies, limiting the generalizability to different populations. Finally, the lack of detailed patient-level data restricted meaningful subgroup analyses and the identification of predictive biomarkers for response.

## 5. Conclusions

In conclusion, the present data do not support the addition of the PD-1/PD-L1 blockade to SOC in the general MM population. Although benefits may exist for specific subgroups, their limited efficacy and increased toxicity highlight the need for future clinical trials that prioritize biomarker-driven designs that test for the expression of different immune checkpoints, Treg abundance and ratios with CD4+/CD8+ effector T cells, markers of pDC activity, and strategic treatment combinations, like combined LAG3, PD-1, and TIGIT inhibition or treatments that target cellular adhesion molecules like VLA-4-mediated drug resistance to define immune checkpoint blockade activity in MM.

## Figures and Tables

**Table 1:** The characteristics of all the included studies.

Studies	Treatment Arms	Control Arm	Number of Patients	Year of Publication
Lesokhin et al. [11]	Isatuximab + cemipilimab Q2W OR Isatuximab + cemipilimab Q4W	Isatuximab	106	2023
KEYNOTE-185 [12]	Pembrolizumab + Lenalidomide + Dexamethasone	Lenalidomide + Dexamethasone	301	2019
KEYNOTE-183 [13]	Pembrolizumab + Pomalidomide + Dexamethasone	Pomalidomide + Dexamethasone	249	2019
CHECKMATE- 039 [14]	Nivolumab + Daratumumab (Cohort A and B) OR Nivolumab + Daratumumab + Pomalidomide + dexamethasone (Cohort A)	Daratumumab	74	2023
CHECKMATE- 602 [15]	Nivolumab + Elotuzumab + Pomalidomide + Dexamethasone OR Nivolumab + Pomalidomide + dexamethasone	Pomalidomide + dexamethasone	170	2024

**Table 2:** The survival statistics for the integrated individual patient data survival analysis.

Group	Sample Size (N)	Median OS (Months)	95% CI (Months)	HR	95% CI (HR)	*p*-Value
No PD/PDL1 treatment	401	24.38	18.78–28.29	1.11	0.87–1.42	0.38
PD/PDL1 treatment	488	22.49	17.38–31.13			

**Table 3:** The survival statistics for all cohorts in each of the five studies.

Study	Arm	PFS-6 Months (%)	OS-6 Months (%)	Median OS (Months, 95% CI)	Median PFS (Months, 95% CI)
Study1	isa	-	-	NR (8.936–NR)	2.89 (1.97–3.81)
Study1	isa/cemi Q2W	-	-	18.96 (6.932–NR)	3.75 (1.97–5.88)
Study1	isa/cemi Q4W	-	-	14.75 (9.04–NR)	3.02 (2.79–5.16)
Keynote-185	Pem/len/dex	82 (73.2–88.1)	87.2 (79.9–92)	Not reached	Not reached
Keynote-185	Len/dex	85 (76.8–90.5)	93.9 (88.1–96.9)	Not reached	Not reached
Keynote-183	Pem/Pom/dex	48 (37–58)	82 (74–88)	Not reached (12.9–NR)	5.6 (3.7–7.5)
Keynote-183	Pom/dex	60 (49–69)	90 (82–95)	15.2 (12.7–NR)	8.4 (5.9–NR)
Checkmate-039	Nivo/Dara (Cohort A)	-	-	-	7.6 (3.2–NA)
Checkmate-039	Nivo/Dara/Pom/dex (Cohort A)	-	-	-	17.0 (NA–NA)
Checkmate-039	Nivo/Dara (Cohort B)	-	-	-	6.6 (4.7–10.3)
Checkmate-039	Dara (Cohort B)	-	-	-	6.6 (3.0–12.8)
Checkmate 602	Nivo/Pom/dex	27 (37.5%)	18 (25%)	-	-
Checkmate 602	Pom/dex	20 (28.6%)	11 (15.7%)	-	-
Checkmate 602	Nivo/Elo/Pom/dex	11 (45.8%)	4 (16.7%)	-	-

## Data Availability

All data used in this paper are present within the clinical trials included in the analysis.

## Supplementary Materials

The following supporting information can be downloaded at https://www.mdpi.com/article/10.3390/cancers17233730/s1. Figure S1: Quality assessment outcomes for all included studies; Figure S2: Leave-one-out plots for all-grade adverse events: (a) immune-related adverse events, (b) endocrine adverse events, (c) anemia, (d) pneumonia, (e) rash, (f) diarrhea; Figure S3: Leave-one-out plots for grade 3/4 adverse events: (a) immune-related adverse events, (b) thrombocytopenia, (c) neutropenia, (d) pneumonia, (e) rash, (f) diarrhea, (g) anemia; Figure S4: Funnel plots for ORR, CR, VGPR, PR, and SD; Figure S5: Funnel plots for incidence of all-grade and grade ≥ 3 immune-related adverse events, neutropenia, thrombocytopenia, and anemia; Figure S6: Funnel plots for incidence of all-grade and grade ≥ 3 diarrhea, endocrine events, pneumonia, and rash.
