# Supplementary figures and images for: Comparison of Standard of Care with or Without a PD-1/PD-L-1 Inhibitor for the Treatment of Multiple Myeloma: A Systematic Review and Meta-Analysis of Phase II and III Randomized Controlled Trials
Source: Cancers (Basel). 2025 Nov 21;17(23):3730. doi: 10.3390/cancers17233730 (PMC12691222; doi:10.3390/cancers17233730)

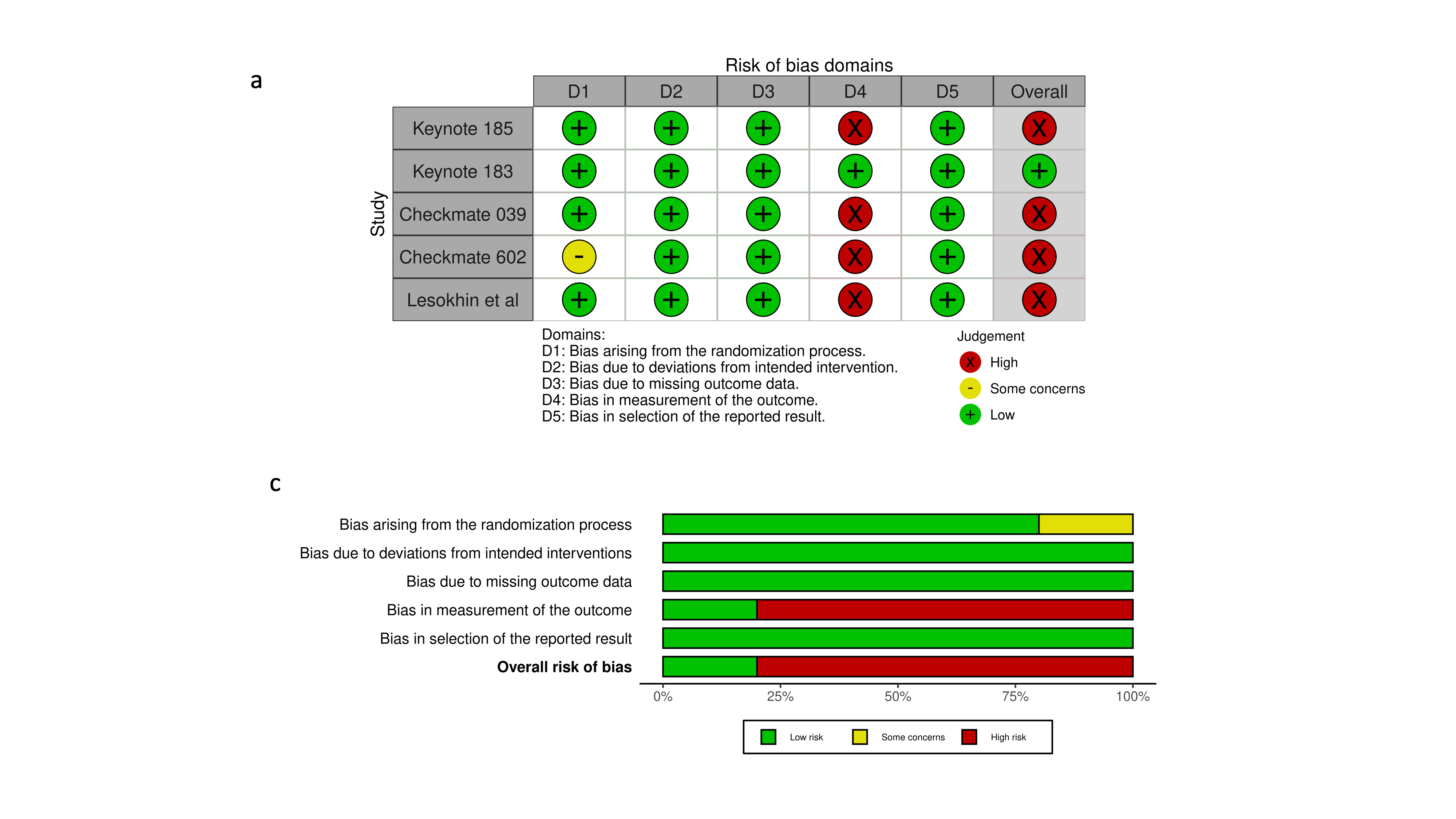

Supplement: Supplementary file 1 [file cancers-17-03730-s001.zip › Supplementary Figure S1.tiff]

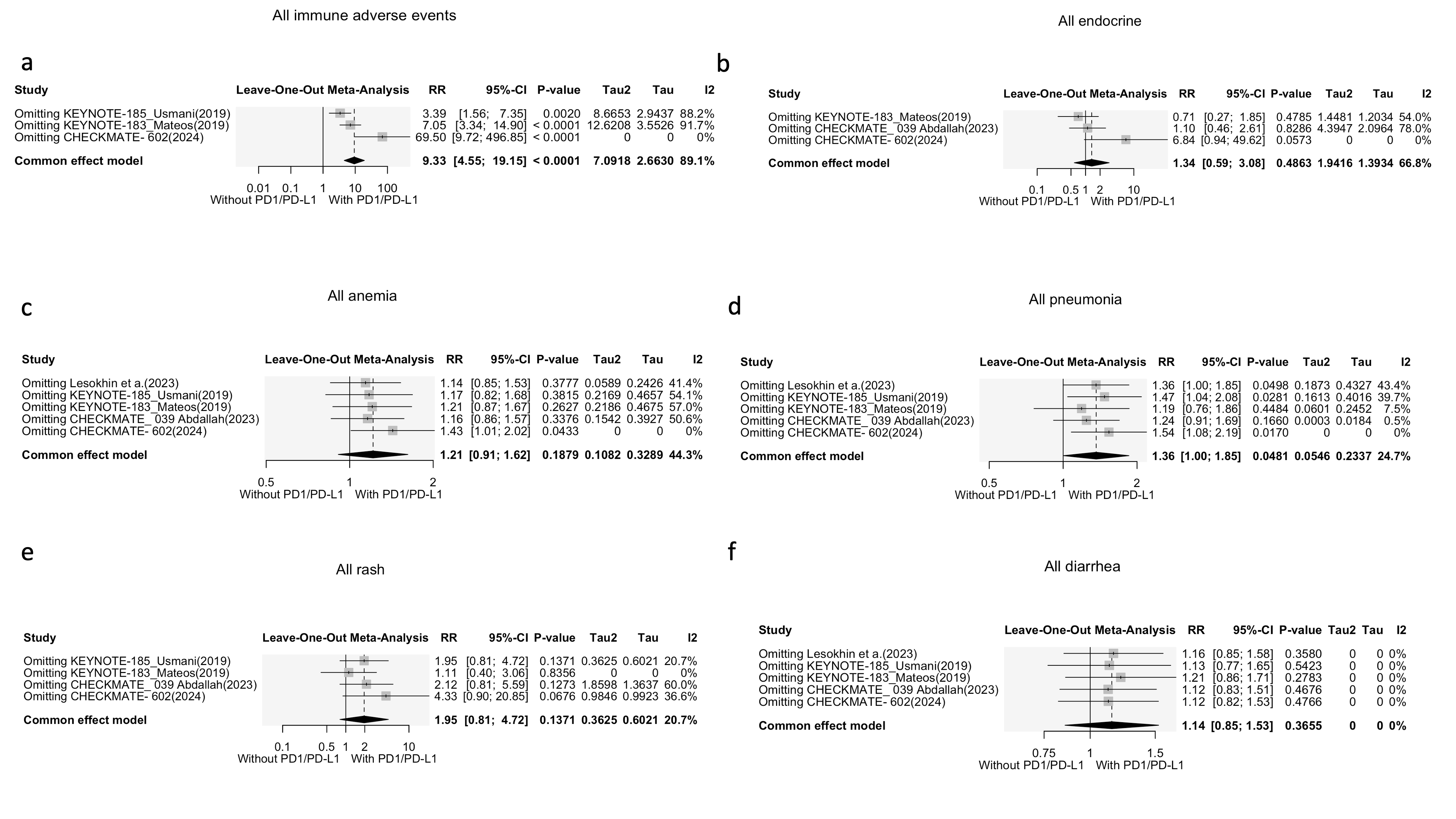

Supplement: Supplementary file 1 [file cancers-17-03730-s001.zip › Supplementary Figure S2.tiff]

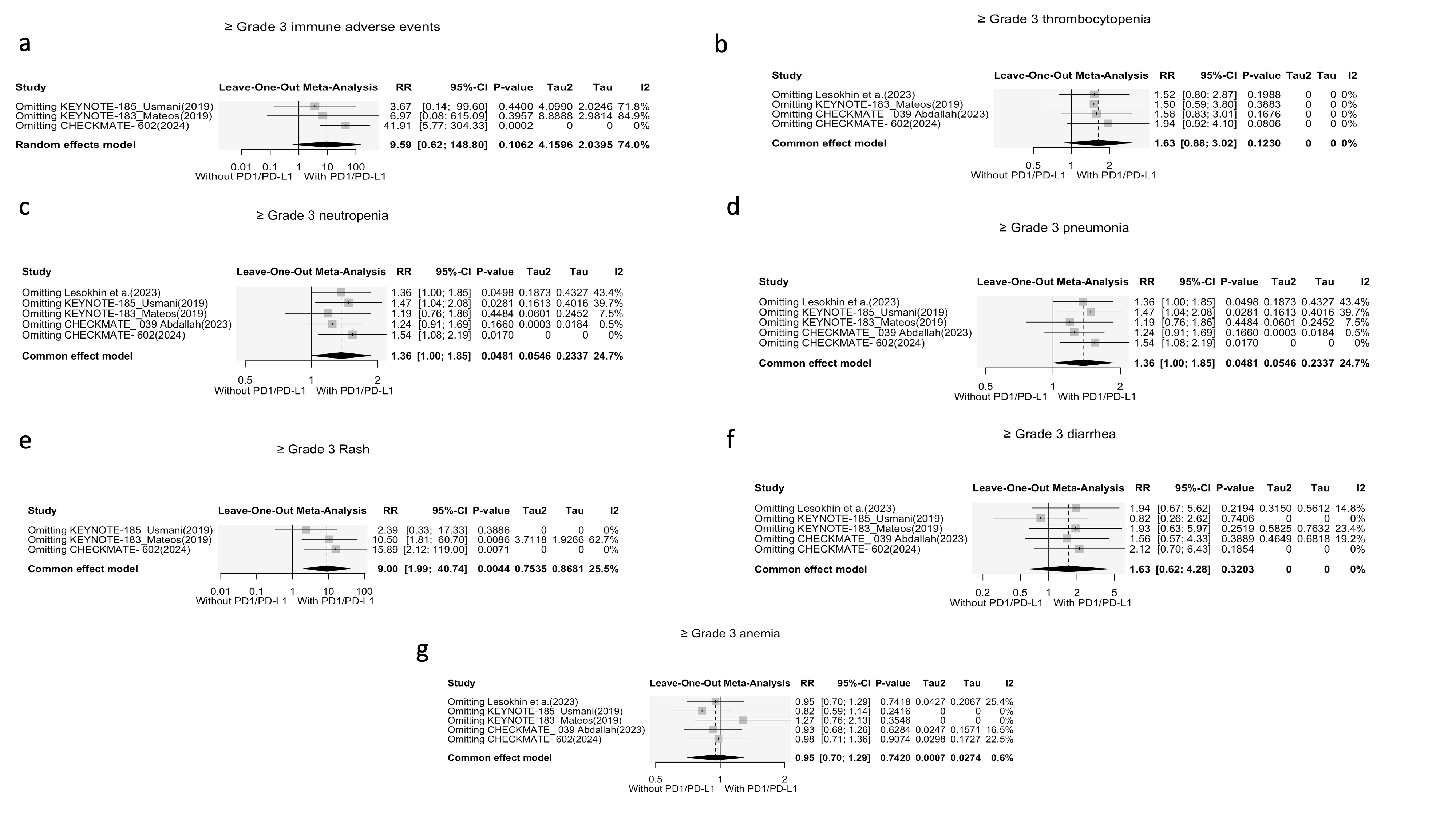

Supplement: Supplementary file 1 [file cancers-17-03730-s001.zip › Supplementary Figure S3.tiff]

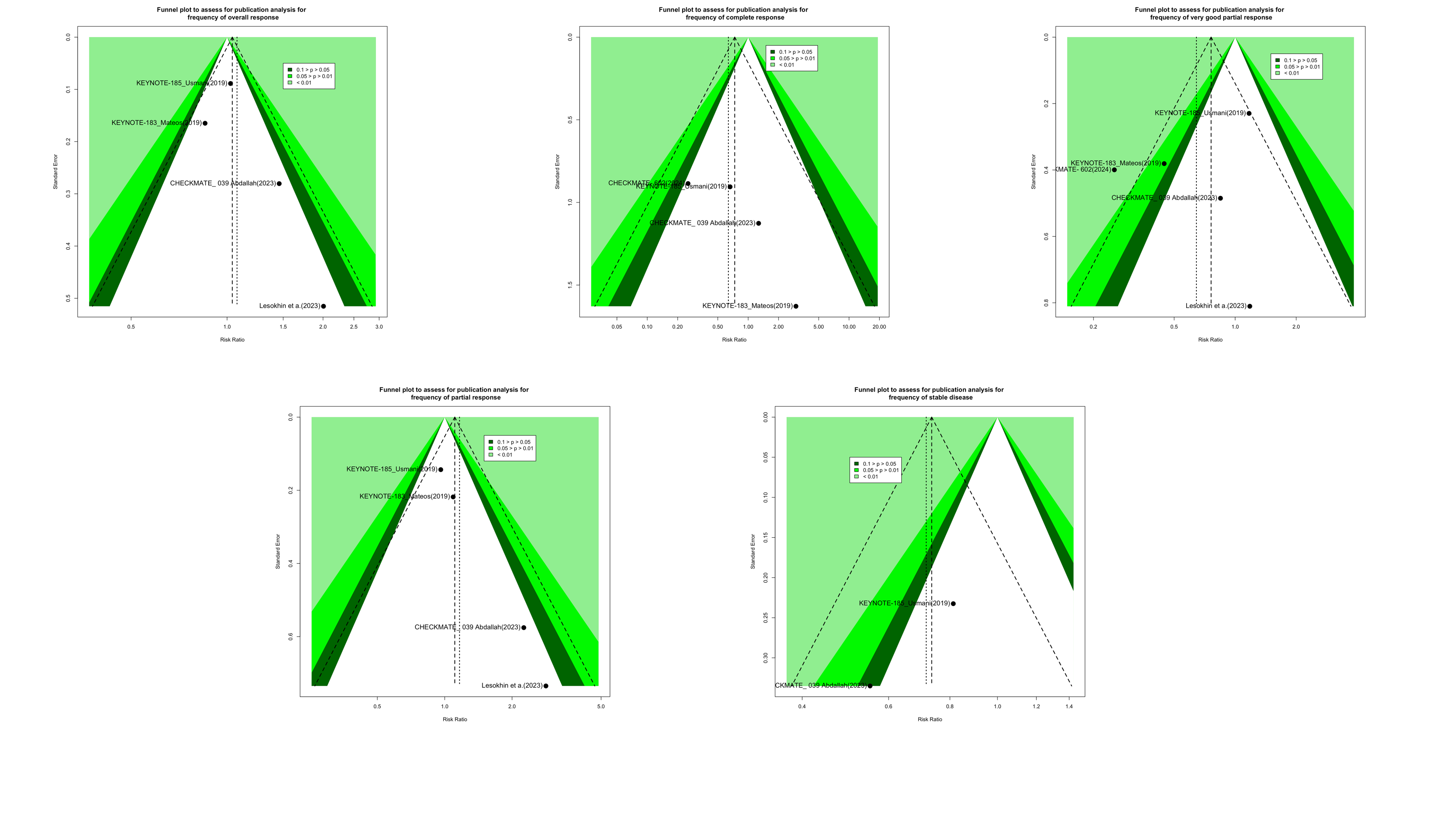

Supplement: Supplementary file 1 [file cancers-17-03730-s001.zip › Supplementary Figure S4.tiff]

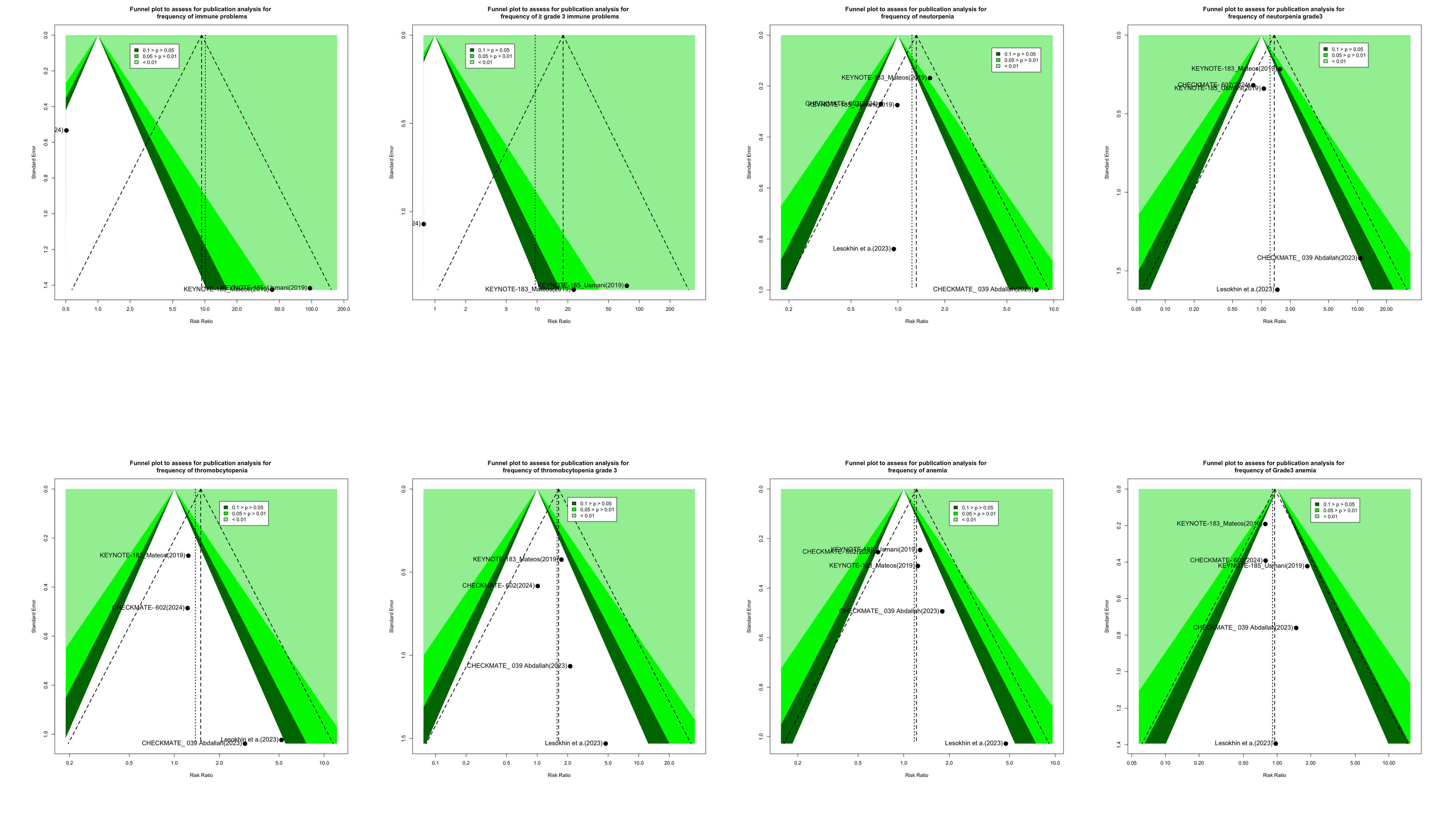

Supplement: Supplementary file 1 [file cancers-17-03730-s001.zip › Supplementary Figure S5.tiff]

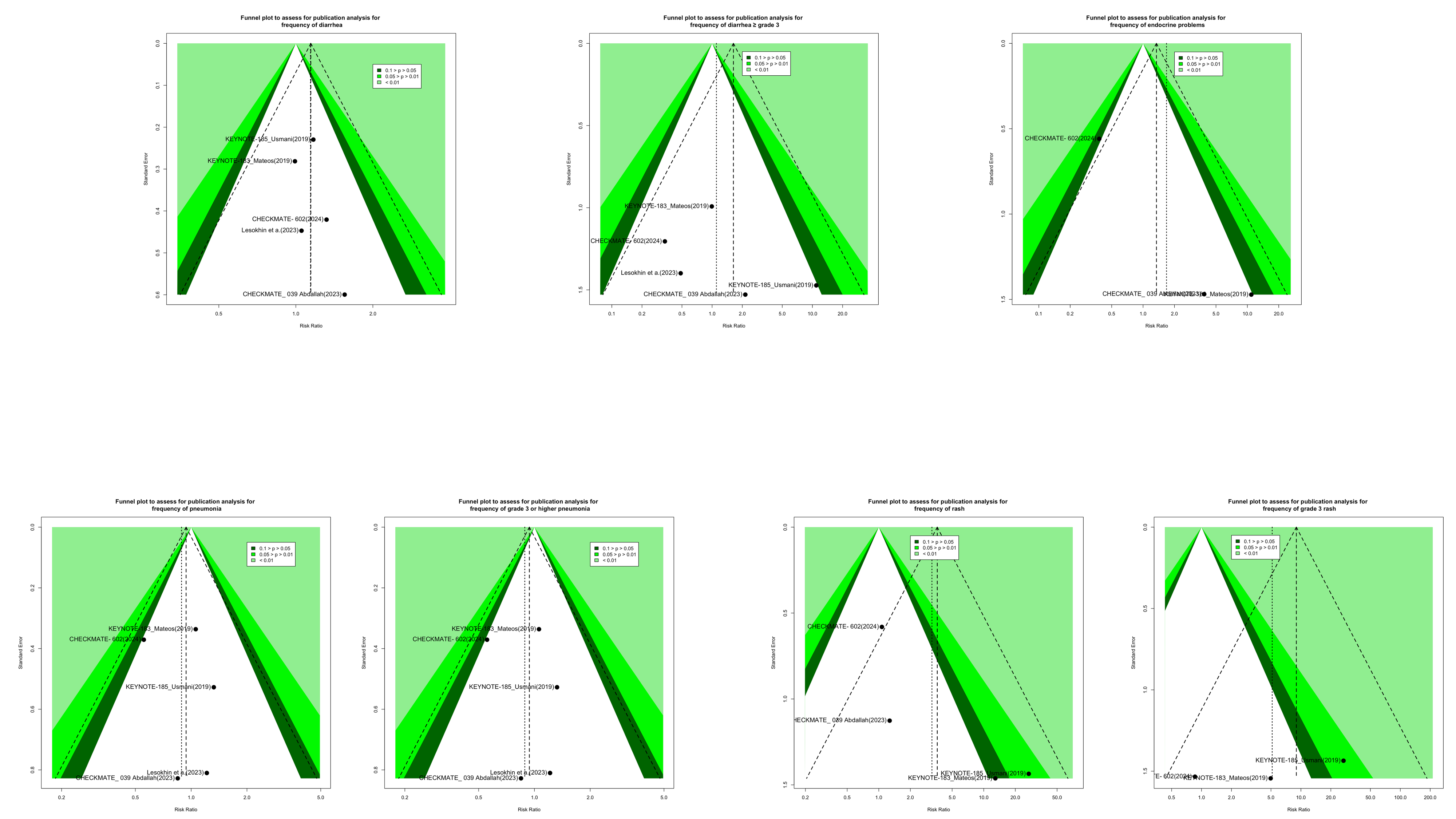

Supplement: Supplementary file 1 [file cancers-17-03730-s001.zip › Supplementary Figure S6.tiff]
